# “I Accept Them, They Accept Me, We Enjoy Our Time Together”: Autistic Adults’ Preferences and Perceptions of Relationships With Other Autistic People

**DOI:** 10.1177/13623613261451898

**Published:** 2026-06-25

**Authors:** Hannah Minnell, Hannah Waddington, Phoebe Jordan, Beth Noble, Chris J. Bowden

**Affiliations:** 1 Te Herenga Waka – Victoria University of Wellington, New Zealand; 2 Access Insights, Wellington, New Zealand

**Keywords:** autism, relationship, autistic adults, perception, preference, qualitative

## Abstract

Autistic adults may have distinct relationship preferences and perceptions compared to non-autistic individuals. The Double Empathy Problem suggests that autistic people often experience greater mutual understanding and connection when interacting with other autistic individuals. While some research has examined these relationships, few studies have focused on a range of relationship types. This study aimed to address this gap by qualitatively exploring how autistic adults prefer and perceive their relationships with other autistic people across friendships, romantic relationships, mentoring/support, employment, and volunteering relationships. We surveyed autistic adults living in Australia and New Zealand to better understand their preferences and perceptions regarding these relationships. Written responses from 142 participants were analysed using a reflexive thematic analysis framework. Many autistic adults valued intersubjectivity, mutual understanding, and acceptance in their relationships with other autistic individuals. Within these relationships, they could be authentic and unmask; however, they also had to consider and balance their relational capacity. In addition, some participants experienced conflict in their relationships due to differences in compatibility. While within-neurotype relationships are beneficial for many autistic individuals, they are not always seamless, and challenges and conflicts can still arise. Like all relationships, autistic–autistic relationships are complex and nuanced.

**Lay Abstract:**

Many autistic people enjoy spending time with other autistic individuals because they feel more comfortable and better understood in these relationships. While some research has explored autistic adults’ relationships with other autistic people, less is known about their preferences and perceptions within these relationships. To learn more, we surveyed autistic adults in Australia and New Zealand about a range of relationship types they may have with other autistic people (i.e. friendships, romantic relationships, mentoring/support, employment, and volunteering relationships). We then analysed written responses from 142 participants using a reflexive thematic analysis to identify themes within the data set. Participants valued intersubjectivity, mutual understanding, acceptance, and a sense of belonging and described being able to unmask and be authentic in these relationships. However, they also had to consider and balance their relational capacity. While shared neurotype was important, other factors like shared interests, values, and emotional compatibility also influenced relationship satisfaction. Some participants also described challenges or conflicts due to differences in compatibility. These findings highlight that many autistic adults have meaningful relationships with other autistic people, challenging a deficit-based perspective. At the same time, they show that shared neurotype alone does not guarantee compatibility. Instead, the Double Empathy Problem should be understood as a spectrum influenced by factors such as shared life experience and social understanding, reminding us that, like all relationships, those between autistic people are complex and nuanced.

## Introduction

Social relationships have been extensively studied in academic literature. However, for the autistic population, who are characterised by having differences in the areas of social communication and interaction, as defined in relation to neuronormative expectations (American Psychiatric Association, 2013/2022), the topic of social relationships has been relatively underexplored. Once assumed to be uninterested in, or incapable of, forming relationships with others, researchers frequently sought to identify and mitigate the perceived “deficits” that autistic individuals experience in the social realm (Sala et al., 2020; Sedgewick & Douglas, 2023). Often, this involved developing and administering interventions to help “improve” social functioning by teaching neuronormative social skills (Bennett et al., 2018). Research has frequently explored the effectiveness of such interventions, measuring social-communication outcomes such as joint attention and social interaction (Wattanawongwan et al., 2023). It is important to note that much of the existing research has focused on improving autistic individuals’ relationships with non-autistic^1^ people, often using neuronormative frameworks of friendship and definitions of “successful social interactions” (Bauminger-Zviely, 2021).

As a result of this deficit-focused discourse, there is a dearth of empirical research exploring autistic individuals’ perceptions and experiences of their social relationships, as these were historically deemed irrelevant (Sala et al., 2020; Sedgewick & Douglas, 2023). However, in recent years, and in alignment with the growth of the neurodiversity movement, contemporary researchers are beginning to challenge these perceptions. It is now known that autistic individuals are interested in having and sustaining meaningful relationships with others (Sedgewick & Douglas, 2023; Strunz et al., 2017). It is also known that the nature and dynamics of autistic relationships may differ from those of non-autistic people (Chan et al., 2023). Thus, some researchers are now advocating to shift away from deficit-focused conceptualisations of autistic relationships towards recognising *differences* in relationship practices relative to neuronormative models of social interaction (Donaldson et al., 2018; Yew et al., 2021). Finke (2023) argues that evidence is robust enough to warrant the exploration of autistic friendships as a distinct group. In addition, there have been calls to reconceptualise the meaning of friendship from an autistic perspective (Brownlow et al., 2015).

There is a small but growing body of research exploring social relationships from the perspectives of autistic individuals. Within this literature, multiple “storylines” about autistic relationships can be identified, reflecting different researchers’ assumptions and gazes. For example, one prominent storyline suggests that many autistic adults prefer online interactions over face-to-face engagement, as online spaces may reduce the impact of social challenges and allow for more comfortable and authentic communication, without the pressure of social norms like eye contact and body language (Forster & Pearson, 2020; Gallup et al., 2016). Another storyline includes the idea that autistic individuals prefer maintaining physical and emotional distance within their friendships (Finke et al., 2019) and basing friendships around shared interests and activities rather than casual socialising (Finke, 2023).

While these findings provide valuable insight, such storylines are often viewed through a neurotypical gaze (McDermott, 2022). Ideas of “closeness” within relationships are often based on neuronormative behavioural norms, such as physical proximity and emotional intensity (Finke et al., 2019). Consequently, when autistic people choose to engage in online spaces or focused activities, these practices are often framed as “maintaining distance” within their relationships. However, these ways of engaging can represent meaningful forms of closeness and allow autistic individuals to feel deeply connected to others (Lizon et al., 2024). When viewed through a neuronormative lens, autistic relational practices that do not align risk being misunderstood or devalued, as ways of being that diverge from neuronormative expectations may become ineligible to the gaze of neuronormative others (McDermott, 2022).

In contrast to this neuronormative storyline, which frames autism through processes of “othering” and distance (McDermott, 2022), another body of research explores the environments and contexts that shape autistic sociality. Many autistic people have experienced a lifetime of difficulties and disappointments due to existing in neurotypical-dominated spaces (Sinclair, 2010). Within these spaces, autistic people may feel confused, misunderstood, or misinterpreted and be expected to maintain a level of participation that can feel draining or overwhelming (Sinclair, 2010). These experiences may be partly explained by differences in dominant forms of sociality across contexts. In neurotypical spaces, social interaction is often organised around socially based sociality, in which social connection is based on group identification, and communication relies on intuitive interpretations of implicit social meanings, which can be tiring and require considerable effort to interpret (Bertilsdotter Rosqvist, 2019).

In contrast, autistic-centred spaces foster interest-based sociality, with social connection centred on shared interests and communication based on genuine interest in a particular topic (Bertilsdotter Rosqvist, 2019). Such spaces provide opportunities for contact, participation, and belonging while accommodating needs that can make neurotypical spaces challenging (Sinclair, 2010). From this perspective, an autistic-centred storyline emphasises that autistic sociality is not defined by distance, but by differently organised forms of connection and participation. Autistic perspectives on sociality therefore contribute to broader understandings of sociality and community while also challenging dominant norms that shape how a social behaviour is evaluated (Bertilsdotter Rosqvist, 2019).

Attentional and energy-based accounts of autistic sociality provide further storylines, most notably through the concept of monotropism (Murray et al., 2005). Monotropism describes a tendency for autistic individuals to focus intensely on a small number of highly arousing interests, in contrast to non-autistic individuals who may distribute attention across a wider range of less intensely arousing interests (Murray et al., 2005). Aspects of monotropism, such as shared interests and a mutual understanding of focused engagement, may foster connections and friendships between autistic people (Black et al., 2024). Monotrophic tendencies may also shape the ways relationships are maintained as they can limit the range of contexts in which relationships are formed or sustained and require high levels of energy when social interaction occurs outside of interest-driven sociality (Bertilsdotter Rosqvist et al., 2023; Black et al., 2024). Therefore, periods of reduced engagement, which are often interpreted as withdrawal or distance, may actually reflect differences in energy allocation rather than reduced relational investment.

Another prominent storyline centres on autistic–autistic relationships, drawing on Milton’s (2012) Double Empathy Problem. This framework posits that communication gaps between autistic and non-autistic individuals arise from differences in social expression, understanding, and experiences. Importantly, these gaps are attributed to bi-directional challenges rather than deficits inherent to the autistic individual (Milton et al., 2018). Consequently, autistic individuals are hypothesised to be better able to empathise and communicate with other autistic people, while non-autistic individuals are better able to empathise and communicate with other non-autistic people (Milton, 2012).

There is a growing body of empirical research to support this framework. Autistic individuals have been found to transfer information more effectively with each other, whereas information transferred between an autistic and a non-autistic person is less effective (Crompton, Ropar, et al., 2020). In addition, autistic individuals report higher levels of interactional rapport when engaging with other autistic people than with non-autistic individuals (Crompton, Sharp, et al., 2020). Observer ratings also indicate greater rapport in autistic–autistic interactions than in those between an autistic and a non-autistic person, or between two non-autistic individuals. This finding suggests that the ease and enjoyment of autistic–autistic interactions is not only subjectively experienced but also externally observable (Crompton, Sharp, et al., 2020). Together, these findings support a reframing of social difficulty as relational and contextual, rather than as an impairment located within the autistic individual. What is considered “difficult” often depends on neuronormative expectations and the social context in which interactions occur (Crompton, Sharp, et al., 2020). Thus, the social differences associated with autism that may be perceived as barriers to forming relationships within neuronormative social contexts may in fact not be barriers at all when both individuals are autistic (Yew et al., 2021).

Research has also explored the role of masking in shaping autistic adults’ social experiences. Masking refers to the conscious and/or unconscious suppression of natural responses and adoption of alternative responses across a range of domains including (but not limited to) behaviour, social interaction, and sensory experiences (Pearson & Rose, 2021). For autistic individuals, masking can include modifying aspects of one’s self, identity, and behaviour in an attempt to appear non-autistic and “blend in” with neuronormative social norms (Bradley et al., 2024; Cage et al., 2022; Miller et al., 2021). These strategies may include hiding autistic traits, using techniques to appear socially “competent” according to neuronormative standards, and actively preventing others from noticing social or communication differences that diverge from these expectations (Hull et al., 2017). While some experiences of masking are shared across all types of people (neurodivergent and neurotypical; Miller et al., 2021; Pryke-Hobbes et al., 2023), autistic people are more likely to report that masking is exhausting, associated with higher levels of stress, and in some cases, linked to suicidal ideation (Scheeren et al., 2025). Masking is also context-dependent, with autistic people more likely to mask in the presence of non-autistic individuals and less likely to do so when alone or with other autistic people (Scheeren et al., 2025). This reduction in masking within autistic–autistic contexts may reflect greater acceptance, shared understanding, and a stronger sense of belonging, which can support more positive autistic social identities (Watts et al., 2025).

While some existing studies provide insights into the relationship preferences and perceptions of autistic adults, this remains an emerging area of research, particularly with respect to qualitative inquiry. To date, much of the literature has focused on friendship, using quantitative approaches to compare the preferences of autistic and non-autistic individuals (e.g. Finke, 2023; Finke et al., 2019). Qualitative studies have similarly centred on perspectives of friendship or romantic relationships, often comparing autistic and non-autistic participants (e.g. Sala et al., 2024; Sedgewick et al., 2019). Such comparison-based approaches, while informative, may also reinforce dominant storylines grounded in neuronormative assumptions about relationships. In addition, little qualitative research has examined autistic–autistic relationships beyond friendship and romance, and there is a notable gap in studies exploring other relationship types, such as mentoring/support, employment, and volunteering relationships.

The present study qualitatively examines how autistic adults perceive and prefer relationships with other autistic people across diverse relational contexts. By moving beyond friendship-focused or comparison-based perspectives, this study examines autistic–autistic relationships as a distinct and meaningful relational group across multiple domains, with the aim of providing a deeper and richer understanding of the nature and importance of these relationships. Specifically, the current study seeks to answer the following research questions:

1. What are autistic adults’ perceptions of their relationships with other autistic people?
2. What are autistic adults’ preferences for forming relationships with other autistic people?

## Method

The data analysed for this article were derived from a larger survey exploring autistic adults’ perceptions and preferences regarding their relationships with other autistic individuals. The survey collected both quantitative and qualitative data. In this paper, we focus on the qualitative responses that were collected using open-text boxes throughout the survey. The quantitative survey results from the larger study will be presented in a separate publication.

### Ethical Approval

Ethical approval was granted by the Victoria University of Wellington Human Ethics Committee (#313733). Participants provided informed consent to participate in the anonymous survey. No identifying information was collected, and participants were asked to exclude identifying information in their responses.

### Recruitment

Participants were recruited primarily through social media (e.g. Facebook), using an advertisement that was shared with autism-specific social media groups (e.g. Autistic Adults Aotearoa NZ, Autistic Self Advocacy Network of Australia and New Zealand) as well as local autism organisations (e.g. Autism New Zealand, Altogether Autism). Participants were also asked to share the study with others who may be eligible and interested in participating.

### Participants

To be eligible to complete the survey, participants needed to be an autistic adult (18 years or older) and currently residing in Australia or New Zealand. We relied on self-reports of autism diagnosis and accepted participants who were either clinically diagnosed or self-identified as autistic in order to accommodate for the significant barriers faced when seeking a clinical diagnosis (Lai & Baron-Cohen, 2015).

Of the 155 autistic adults who completed the online survey, 142 provided usable qualitative responses, which have been analysed in the current study. We detected a high number of bot-generated and fraudulent responses, likely due to the voucher draw that participants could opt to enter upon survey completion. In total, 676 fake responses were removed before analysis. These were identified based on highly similar or identical answers submitted simultaneously (suggesting automated entries), very short completion times (with identical times indicating bot activity), identical IP addresses, low CAPTCHA scores, and high fraud and duplicate scores flagged by Qualtrics security measures. To further mitigate bot activity, the survey was temporarily closed several times and reopened after a few hours.

Demographic information was collected to characterise the sample, including age, self-reported gender, self-reported sexual orientation, education, and employment. Most participants were female, Australian or New Zealand European, and under the age of 50. Full demographic details are provided in Table 1.

**Table 1.** Participant Demographic Information.

Participant characteristics	*n* (%)
Country of residence	
Australia	44 (31)
New Zealand	98 (69)
Age (years)	
18–24	29 (20)
25–30	31 (22)
31–35	16 (11)
36–40	23 (16)
41–45	18 (13)
46–50	9 (6)
51–55	8 (6)
56–60	2 (1)
60–65	2 (1)
65+	4 (3)
Gender	
Male	30 (21)
Female	76 (54)
Gender diverse	36 (25)
Sexuality^a^	
Heterosexual	61 (43)
Bisexual	33 (23)
Homosexual	9 (6)
Asexual	4 (3)
Queer	16 (11)
Pansexual	13 (9)
Demisexual	3 (2)
Sexually fluid	1 (1)
Questioning/unsure	3 (2)
Prefer not to say/unlabelled	4 (3)
Ethnicity^a^	
Australian/New Zealand European	125 (88)
New Zealand Māori	12 (8)
Cook Island Māori	1 (1)
Aboriginal or Torres Strait Islander	1 (1)
Indian	3 (2)
Chinese	2 (1)
Samoan	1 (1)
Other ethnicities^b^	14 (10)
Prefer not to say	1 (1)
Highest level of formal education	
Primary/intermediate	3 (2)
College/High school	24 (17)
Diploma/Certificate	6 (4)
Bachelors/Undergraduate university degree	42 (30)
Postgraduate university degree	43 (30)
Trade/technical/vocational training	23 (16)
Other (“Special Education Primary School”)	1 (1)
Diagnostic status	
Officially diagnosed	108 (76)
Self-identified	31 (22)
Prefer not to say	3 (2)
Total	142 (100)

a Some participants made multiple selections.

b Other ethnicities include: British, European, Fijian, Irish, Malaysian, Middle Eastern, Puerto Rican, South African, Southeast Asian, North American, U.S. European.

### Materials

Participants completed an online survey that was comprised of four sections: (a) demographic information, (b) questions pertaining to their current relationships, (c) questions regarding future relationships, and (d) questions concerning past relationships. A copy of the survey is provided in the Supplemental Material.

While most survey questions were quantitative, each section included several qualitative questions that allowed participants to provide open-text responses. Table 2 provides examples of the qualitative questions used in the survey, along with an overview of the quantitative items. After each set of quantitative questions, participants were given the option to elaborate on their responses using an open-text box with the prompt: “*If you like, you can tell us more about why you selected your response.*” Data for this study were collected from these answers, along with the open-ended questions provided in Table 2. The survey explicitly focused on non-familial relationships. Participants were provided with examples of different relationship types they may have with other autistic people, and familial relationships were not included as response options. During data cleaning, any responses mentioning family relationships, whether qualitative or quantitative, were removed as these were not relevant to the current study. Upon completing the survey, participants could opt to enter a voucher draw for a chance to win 1 of 20 $25 NZD Prezzy Virtual Cards.

**Table 2.** Examples of Questions in Each Survey Section.

Survey section	Overview of closed survey questions	Examples of open survey questions
Section 1: Current relationships questions	Section 1 explored participants’ current relationships with other autistic individuals, including relationship types (e.g. friendships, employment, romantic, mentoring), satisfaction levels (rated on a 5-point scale), ways of connecting (e.g. doing activities, texting, online gaming, being intimate), and connection frequency (from more than once a week to less than once a month)	“Why would you like to improve the quality of the relationships you have with other autistic people?” “Why would you not like to improve the quality of the relationships you have with other autistic people?”
Section 2: Future relationships questions	In Section 2, participants rated their interest in forming different types of relationships on a 3-point scale and provided preferences for connection methods, the number of people they would like to connect with, and frequency of connection.	“Why would you like to form more relationships with other autistic people?” “Why would you not like to form relationships with other autistic people at the moment?”
Section 3: Past relationships questions	Section 3 contained retrospective questions about relationships participants may have wanted to form with other autistic people when they were children or teenagers, including relationship type, number of people, and frequency of connection.	“Why would you have wanted to form more relationships with other autistic children/teenagers when you were a child or teenager?” “Why would you have wanted to improve the quality of the relationships you had with other autistic people when you were a child or teenager?” “Why would you have not wanted to improve the quality of your relationships with other autistic people when you were a child or teenager?”

### Procedure

We used the online survey tool Qualtrics to collect data. The survey was open for 6 weeks from June to July 2024. Prior to beginning the survey, participants were provided with a plain language information sheet which described the study, gave an estimated completion time, and had contact details of the research team. Informed consent was obtained prior to the commencement of the survey. Demographic information was collected first. Following this, participants were directed to the survey where they provided both quantitative and qualitative responses. As the qualitative responses were already written in text format, no transcription was needed. The voucher draw was conducted in the week following survey closure. Participants were randomly selected and contacted via the email address they provided in a separate, unlinked survey to maintain anonymity.

### Survey Development and Participatory Methods

This study was co-designed alongside two autistic adults (one female and one non-binary) who acted as advisors on the project. As the lead author is non-autistic, she collaborated closely with these advisors to help mitigate the influence of a non-autistic gaze. The first author met with the advisors several times to provide input and feedback on the design of the survey. Following each meeting, the advisors were given a $50 NZD supermarket voucher to thank them for their time and expertise. In addition, an autistic male in Australia provided written feedback on the survey draft (to gain a male perspective) and was compensated with $50 AUD Prezee eGift Card. The survey was piloted by PJ, an autistic member of the research team, who also helped with online survey recruitment and data analysis. PJ was an employee of Victoria University of Wellington.

### Positionality Statement

The first author is a female, New Zealand European, non-autistic PhD candidate undertaking a co-designed doctoral project exploring relationships within the autistic adult community. While not autistic, she is formally diagnosed with attention-deficit hyperactivity disorder (ADHD), and her developing understanding of her own neurodivergence and its influence on relationships has shaped her interest in this field. This ADHD positionality is acknowledged as shaping how the lead researcher approached the project, bringing an ADHD-informed “gaze” that may have influenced how social connection, compatibility, and relational experiences were viewed and interpreted. The first author is supervised by the second and last authors who are neurotypical academics in the field of educational psychology.

The first author co-designed this project in collaboration with the third and fourth authors, who are autistic. Their lived experience and expertise were deeply valued and informed all stages of the research project. As such, this research was shaped through the interaction of multiple perspectives, including autistic, ADHD, and neurotypical lenses, each of which may influence how relationships are conceptualised and interpreted. All authors approach neurodiversity as a brain-based difference rather than an inherent deficit and acknowledge that their experiences, values, and knowledge inevitably shape the research process and the interpretations presented in this article. This reflexive awareness underpins a neurodiversity-affirming theoretical stance and a commitment to expanding how relationships are conceptualised beyond traditional, neurotypical assumptions.

### Data Analysis

Data analysis followed the procedure for reflexive thematic analysis (RTA), as outlined by Braun and Clarke (2021). RTA was chosen as it is a flexible, inductive approach that does not rely on existing frameworks to analyse data. Rather, new knowledge is created through the process, which is suitable for this emerging and under-researched topic. Reflexive TA involves (a) familiarisation with the data; (b) generating initial codes; (c) searching for, or identifying, themes as central organising concepts that represent shared conceptual patterns across the data; (d) reviewing themes; (e) refining, defining, and naming themes; and (f) report production (Braun & Clarke, 2022).

It is important to acknowledge that the research team brought overlapping perspectives shaped by members’ neurotype, lived experience, and research backgrounds to the current project. The first and last authors led the initial thematic analysis, approaching the process from the perspective of non-autistic educational psychology researchers. To account for the potential influence of non-autistic perspectives on interpretation, data were analysed primarily at the semantic level, with a focus on participants’ explicit statements rather than applying theoretical frameworks to infer underlying meaning (Braun & Clarke, 2022). This approach was chosen to reduce the risk of misinterpreting autistic experiences through a non-autistic lens and to prioritise participants’ own meanings.

All survey responses were downloaded from Qualtrics, and qualitative responses were collated into a single document for analysis. Participant quotations were retained verbatim and labelled using identification numbers (e.g. P1). The first author read through the dataset multiple times to achieve familiarisation and generated initial codes reflecting patterns and points of interest within the data. She then examined the dataset for areas of shared meaning, grouping responses based on perceived similarities. Initial themes were generated using participants’ own language (e.g. “understanding”).

The first and last authors met fortnightly between October 2024 and March 2025 for analytic discussions and to collaboratively develop and refine themes. Several draft versions of the findings section were then reviewed by the third author, an autistic researcher, who checked the themes for resonance and contributed to their refinement. She provided suggestions for renaming some themes and sub-themes; for example, the first author had originally titled Theme 4 as “personal preferences” to reflect participants’ descriptions of traits and characteristics they considered when forming relationships. PJ suggested that this theme better captured the idea of “compatibility beyond neurotype,” which fed into the renaming of the theme “Compatibility and Fit.” PJ also assisted in developing the narrative used to weave participant quotations together and offered guidance on key areas of emphasis for the Discussion section.

The fourth author, an autistic adult and survey co-designer, also provided feedback on the draft manuscript, along with the second author, the lead author’s primary PhD supervisor. Throughout the analytic process, the first author maintained a reflexive journal and analytic memos to support ongoing reflexive engagement with the data. Regular discussions within the research team were used to review analytic decisions and ensure interpretations remained grounded in participants’ perspectives.

## Results

Participants discussed their perceptions and preferences regarding relationships with other autistic people. Four main themes were constructed from the data:

- *Intersubjectivity and Mutual Understanding*
- *Refuge for the Authentic Self*
- *Labour and Limits of Connection*
- *Compatibility and Fit*

**Figure 1.**
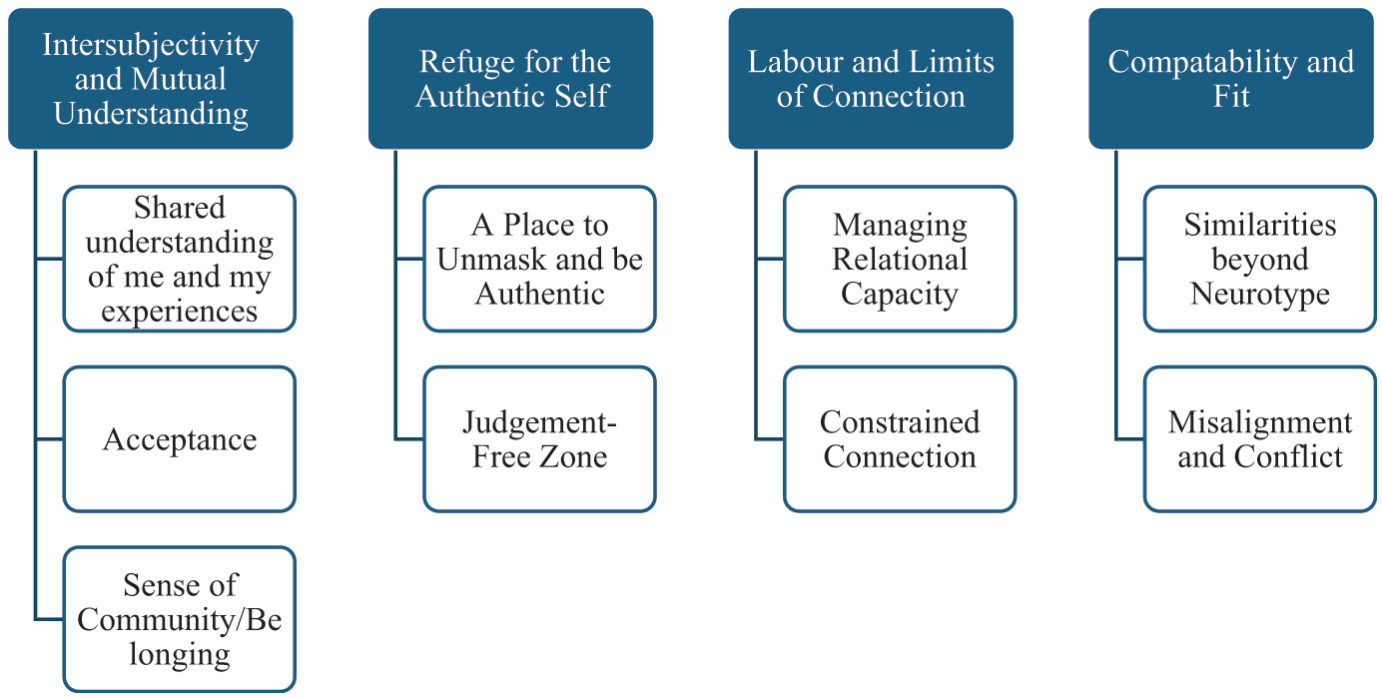
Structure of themes and sub-themes.

Each theme comprises several sub-themes, as outlined in Figure 1.

### Theme 1: Intersubjectivity and Mutual Understanding

A key theme across the participants (P.) descriptions of what they valued in their relationships with other autistic people was intersubjectivity. Participants valued relationships characterised by mutual recognition and expressed a desire for connections with people who recognised and understood their worldview, ways of making sense of the world, experiences, and intentions. Intersubjectivity and mutual understanding contributed to social attunement, relational safety, acceptance, and a sense of belonging and community.

#### Sub-theme 1: Shared Understanding of Me and My Experiences

Participants valued having a deep understanding and connection in their relationships with other autistic individuals: “*I feel that my autistic partner understands me in a way that no one else in my life does or has before* . . .” (p. 79). This shared understanding was often missing in their relationships with non-autistic people: “*They [autistic people] understand my unique experiences, struggles, and strengths in a way that neurotypical people often cannot.*” (p. 58). Participants described how autistic individuals were the only ones who truly “got” them or were on the same “wavelength.” They valued the mutual understanding and validation that came from relationships with others who could relate to their experiences: “*I find that connecting with other autistic individuals fosters a deeper sense of understanding and camaraderie, as we share similar experiences and perspectives.*” (p. 58). For some, shared understanding was also the foundation for emotional support: “*Communicating with other autistic individuals would allow me to receive deeper understanding and empathy. They would be able to truly comprehend my experiences, struggles, and unique thought processes, which is incredibly important to me.*” (p. 102).

Several participants believed that shared neurotype could be beneficial in romantic relationships, for fostering greater mutual understanding: “*Maybe if the right person for me romantically was autistic we might understand each other more*.” (p. 57). Some wanted connections with other autistic individuals who could understand the challenges they faced in the workplace and with those who could offer meaningful support: “*To feel more understood and find a broader support network and community. To have a mentor who understands me and the challenges I am likely to face in my career*.” (p. 79).

#### Sub-theme 1.2: Acceptance

Autistic–autistic relationships were valued because they provided acceptance. Participants appreciated how other autistic people “*don’t make me feel weird or awkward*” (p. 41), and they enjoyed spending time with others who inherently understood their needs: “*It’s nice to be with people who ‘get’ that it’s okay to be different and be ourselves and there is an acceptance that we may need some accommodating for sometimes and that’s okay*.” (p. 132). Mutual acceptance of each other’s differences, ways of being, and personal perspectives made these relationships feel easy: “*I accept them, they accept me, we enjoy our time together. We can agree-to-disagree where we share different views. It’s easy.*” (p. 34).

Relationships with others of the same neurotype were viewed as fostering self-acceptance, greater authenticity, and a sense of belonging. Spending time with fellow autistic individuals enabled participants to relax and better accept their own autism: “*I would very much like to have mentoring relationships in the context of my employment as I feel it will make me feel more supported and accepting of myself. I think it will also make me feel more understood at work*.” (p. 130).

Some participants wanted to form more relationships with other autistic people to experience more of this acceptance. For example, one participant explained, “*A meaningful relationship with a female with autism would be great because I wouldn’t need to apologize all the time or explain my behavioral responses*.” (p. 67). This sentiment underscores how these connections reduce the social burden often felt in non-autistic relationships.

#### Sub-theme 1.3: Sense of Community/Belonging

Several participants described feeling isolated in neurotypical spaces and saw relationships with other autistic individuals as a way to foster a sense of community and belonging. Many said they felt as though they had never “fitted in” with a neurotypical society and spent much of their lives feeling lonely, excluded, and a lack of belonging: “*Definitely to reduce loneliness and create that feeling of community, like I belong here, I’m not a freak, I’m not alone in the world. There are people here who actually understand my experiences first hand*.” (p. 23). Some sought kinship and attachment through a shared identity: “*. . . to be part of a community of people who understand what it’s like to be autistic. For most of my life I’ve felt like I don’t belong . . .*” (p. 6).

Others wanted to help fellow autistic individuals build relationships and connect with the wider autistic community, recognising the challenges of isolation firsthand: “*I know how hard life can be when you are autistic, and I want to be able to show up for my autistic peers.*” (p. 33). Some were passionate about helping others form meaningful networks and connections: “. . . *being part of this wonderful autistic community and helping others find and connect with this community so they too do not have to be isolated from their autistic culture.*” (p. 132). Several participants were particularly interested in mentoring: “*Mentoring would interest me as it can be hard to navigate, and I’d be glad to help those going through struggles I’ve experienced myself*” (p. 50).

### Theme 2: Refuge for the Authentic Self

A central theme in participants’ descriptions of autistic–autistic relationships was that these relationships offered a social space and context in which they could be their authentic selves. These relationships provided a space where they could unmask, connect genuinely, and experience acceptance without fear of judgement.

#### Sub-theme 2.1: A Place to Unmask and Be Authentic

Participants described being able to unmask and be authentic which provided relief from the fatigue and pressures of having to present or “act” a certain way in neurotypical spaces: “*I spend a lot of time acting, especially at work around neurotypical people. I want to be free. I think it is easier to let go in front of other autistics*.” (p. 37). Spending time with other autistic people was easy and comfortable; these interactions felt more “natural” and required less effort: “*I can usually tell someone is likely autistic quite quickly. I feel I can unmask around them and them around me*.” (p. 85).

Within autistic–autistic relationships, participants could express themselves openly:

“*I have two autistic friends and two (suspected) ADHD friends. When I’m with them, I don’t feel like I’m trying to be somebody else. It’s easy to speak up about my needs and wants, and for them to do the same with me. The friendships are all direct and open and full of compassion and understanding. I very rarely doubt that these people like me or want to be around me*.” (p. 129). In contrast, many participants did not experience this comfort, safety, and acceptance in their relationships with non-autistic people, noting how “*many neurotypicals don’t give me the same vibes*.” (p. 58).

#### Sub-theme 2.2: Judgement-Free Zone

Participants described feelings of security and relief in autistic–autistic relationships due to the absence of judgement. While many felt scrutinised or misunderstood in non-autistic relationships, connections with other autistic people created a “*judgement-free zone*” (p. 65) where they could be their authentic selves. As one participant shared: “*I can unmask easier around them and be less socially anxious because they won’t judge me*.” (p. 16).

Safety was important and experienced around others who understood some of the hurt and challenges they experienced: “*We really get on as we are all honest and non-judgemental and aware of trauma in the past so recognise we must communicate clearly*” (p. 145). Participants also felt that autistic people did not minimise or dismiss their struggles: “*To have people to talk to who understands my experience and won’t judge me. The issues I face as an autistic person can be hard for allistic* [non-autistic] *people to understand and take seriously it seems*.” (p. 112).

### Theme 3: Labour and Limits of Connections

A key theme within participants’ descriptions of autistic–autistic relationships was that these relationships (like others) require significant relational, cognitive, and social labour and effort to establish and maintain. Energy, interest, and time were things participants needed to invest in relationships, but there were also environmental and health-related barriers that made forming, negotiating, and sustaining relationships more challenging.

#### Sub-theme 3.1: Managing Relational Capacity

Participants’ capacity for relationships was not infinite and was enabled or constrained by the amount of energy, interest, and time they had to invest. While many valued having relationships with other autistic people, the need to balance social capacity was often acknowledged: “*I would like to see my autistic friends more, but it would have to be in a way that was not taxing on our energy*.” (p. 21). While autistic–autistic relationships were often easier and required less labour, they still required a significant amount of emotional and physical energy. Many described relationships as being “exhausting”: “*I don’t know how to sustain deeper friendships as they can be exhausting. Autistic relationships are less exhausting but still tiring*.” (p. 95).

Some participants described having limited energy and motivation to plan, make decisions, track responsibilities/commitments, and engage in their current relationships: “*I’m a bit low at the moment so haven’t had the spoons*^2^ *or motivation to do much socialisation. Would be nice to be a bit healthier and nurture some of my relationships*.” (p. 88). Others noted that burnout and social exhaustion limited how often they could engage with friends: “*I already have a level of closeness and frequency that I am happy with, but to see these two friends more often would be draining*.” (p. 15). Having insufficient energy meant some participants had limited interest in forming new relationships: “*The main factor that reduces my interest in forming additional relationships is the limited number of spoons I have to form any new relationships, autistic or allistic*.” (p. 33).

Limited capacity was also shaped by participants’ energy levels, along with time and availability: “*At the moment, I don’t have the time for more friends. All my slots are taken*.” (p. 118). This lack of capacity sometimes resulted in social withdrawal and avoidance, with one participant explaining how they are “*mostly people adverse at the moment*” (p. 32). Limited capacity to engage in future relationships with anyone, regardless of neurotype, was also a challenge due to the emotional labour required: “*I just want to specify that the reason why I am only somewhat interested in having romantic relationships with other autistic people is because I don’t have the currently have the capacity for engaging in new romantic relationships with anyone; regardless of neurotype*.” (p. 107).

#### Sub-theme 3.2: Constrained Connection

Forming, accessing, and maintaining relationships was impacted by factors other than labour for some participants. Participation and autonomy to engage in social interaction were shaped by structural constraints (e.g. schedules, time, responsibilities), cognitive constraints (e.g. limits of energy and attention), relational constraints (e.g. time scarcity, emotional availability), resource-linked constraints (e.g. money, transport), and contextual constraints (e.g. situations):“*I find timing and scheduling and initiating contact really hard, especially as many of them have ADHD. Physical proximity, transport, and accessible spaces can be barriers, as well as general energy levels*.” (p. 73). Chronic health issues and a lack of autistic people in the community sometimes prevented in-person meetings and hindered opportunities to socialise: “*Because of my chronic illnesses, I don’t get out much, and where I live, there is not much going on solely for finding adult autistic friendships. I also find it hard to build friendships with people online*.” (p. 126).

### Theme 4: Compatibility and Fit

The final theme focuses on the idea of compatibility and perceived fit. Participants valued and sought relationships where there was alignment, considering not only surface-level similarities but deeper factors such as shared values, goals, needs, and personalities, which often extended beyond shared neurotype. Some preferred to form relationships based on similarity, including shared interests, identity, experiences, and common values as this helped build intersubjectivity and mutual connection. However, they also considered potential for conflict, differences in interests and opinions, ability to regulate, and capacity to relate well when forming relationships.

#### Sub-theme 4.1: Similarities Beyond Neurotype

Some participants preferred relationships with fellow autistic individuals, valuing the sense of similarity and shared experience: “. . . *I would rather whanaungatanga* [create connections with] *with tangata takiwatanga me aroreretini* [people with autism and ADHD] *than neurotypicals - for self-care reasons*.” (p. 32). Others emphasised how factors such as physical proximity, compatibility, shared interests, and personality traits mattered more than neurotype alone:

*I think other criteria would be more important to me than their neurotype . . . if another person is autistic, this doesn’t mean I’ll automatically have a lot in common with them. I would look for someone with the same interests instead, who is in my local area, and who is kind*. (p. 118).

Demographic and cultural backgrounds also influenced relational compatibility. Some participants reported finding it easier to relate to those with shared life experiences: “. . . *I find it hard to relate to people who are not belonging to a minority or in lower SES circumstances. This is the major factor in my dissatisfaction among work plus volunteer-based relationships.*” (p. 32).

Not all autistic individuals felt inherently compatible with other autistic people. Some believed shared neurotype was not enough to form meaningful relationships: “*I don’t necessarily need to have more or better relationships with other autistic people because I don’t always have much in common with them*” (p. 122). One participant felt that certain subgroups of autistic individuals could be unexpectedly critical of social missteps: “*Some autistic people, particularly the young white middle class self-identifying assigned female at birth ones, can be surprisingly rude and judgmental of social slip ups from autistic males.*” (p. 10).

#### Sub-theme 4.2: Misalignment and Conflict

Compatibility challenges and mismatches within autistic–autistic relationships were concerns. Several participants expressed frustration with autistic people having a fixed mind-set: “. . . *many autistic people frankly annoy me with their rigidity of thinking and total inability to hold normal conversations* . . .” (p. 122). Non-complementary similarities were also an issue, for example, mutual “rigid thinking” could make it difficult to resolve disagreements: “*Honesty, a lot of autistic people just butt heads and we are so black and white in our thinking it can be difficult to move on from a disagreement.*” (p. 40).

Some participants shared that relationships with other autistic people could also be challenging because of their perceived negative outlook and tendency to be overly political:

“*I don’t like most other autistic people. I find they complain too much or make autism their entire personality. Most of them I’ve spoken to are overly political and get incredibly angry if anyone has a differing opinion.*” (p. 53).

Others recognised that their own intolerance and lack of patience could be part of the problem: “I don’t get along with most autistic people due to how intolerant I am of others. I also do not agree with opinions most of them hold. I’m very fussy with friendships and tend to have a majority of my friends be neurotypical.” (p. 53).

Managing conflict and disagreements while preserving the relationship could be difficult. Some emphasised the potential value of external support in resolving conflict: “*It would be useful to have mediators to help guide us through on occasion so we get positive outcomes*.” (p. 132).

## Discussion

This study aimed to explore autistic adults’ preferences and perceptions of their relationships with other autistic people across multiple relational contexts, using RTA. Social relationships are an important, yet understudied, topic within the field of autism research (Crompton, Hallett, Ropar, et al., 2020). Much of the previously published research has explored autistic peoples’ relationships with non-autistic individuals or compared and/or contrasted autistic individuals’ friendships and romantic relationships to those of the neurotypical population. Consequently, there is a dearth of research exploring other types of autistic–autistic relationships such as mentoring/support, employment, and volunteering relationships.

Our findings indicate many autistic adults valued intersubjectivity, mutual understanding, acceptance, and a sense of belonging in their relationships with other autistic people, where they could be authentic, unmask, and felt free from judgement. Their capacity for relationships depended on their available energy, interest, and time, while environmental and health-related barriers posed challenges. Relationship preferences extended beyond neurotype, with some prioritising similarity in interests, identity, experiences, and values. Participants also considered factors like potential conflict, differences in interests and opinions, emotional regulation, and the ability to relate well when forming relationships.

Our results align with previous research showing that autistic–autistic interactions differ in important ways from neuronormative assumptions about relational practices (Chan et al., 2023; Crompton, Hallett, Ropar, et al., 2020; Watts et al., 2025). Many participants in the current study reported feeling better understood, more accepted, less judged, and more able to be authentic in their relationships with other autistic individuals. They also described these relationships as more enjoyable and “easier” than those with neurotypical people. This finding supports the Double Empathy Problem, which suggests that difficulties in understanding and empathy between autistic and non-autistic individuals stem from differing world experiences rather than a communication deficit in autistic people (Milton, 2012). As a result, both groups experience mutual difficulty understanding and empathising each other. This is echoed in our findings, which add to the growing body of empirical evidence supporting this framework.

We found that many autistic adults desired deep, meaningful relationships. This finding contrasts with distance-focused storylines, where autistic people are often characterised as preferring both physical and emotional distances within their relationships or struggling with intimacy (e.g. Dean et al., 2017; Finke et al., 2019; Girardi et al., 2021). Instead, our findings suggest that many autistic adults do seek closeness, though their ways of expressing it may differ from neuronormative notions of closeness. It is possible that previous research has misinterpreted autistic social behaviours or defined closeness using assumptions that do not reflect autistic preferences.

There is increasing evidence to suggest that autistic people approach and define relationships differently, view aspects of those relationships differently (e.g. connection, interaction, communication), and use a different set of social skills compared to neurotypical individuals (Chan et al., 2023; O’Hagan & Hebron, 2017). For example, autistic people may foster unique forms of closeness through special interests, autistic “social play,” and a mutual understanding of each other’s sensory needs and communication styles (Black et al., 2024; Cummins et al., 2020; Long, 2025; Pritchard-Rowe et al., 2024). Such methods of engagement are characteristic of autistic-centred spaces, where social connection often emerges around shared interests (Bertilsdotter Rosqvist, 2019). While some neuronormative researchers may perceive these preferences as signalling a greater distance in relationships, they can in fact represent meaningful and fulfilling forms of connection for autistic people.

We found that some participants were able to unmask in their relationships with other autistic people. This aligns with prior research suggesting that autistic people may find it easier to unmask, at least partially, in interactions with other autistic people (Khudiakova et al., 2025; Scheeren et al., 2025; Watts et al., 2025). However, recent research suggests that this reduced need for masking may be shaped more by perceptions of psychological safety and communication style “fit” than by shared neurotype alone, and not all autistic individuals are experienced as safe or inherently compatible (Khudiakova et al., 2025). This is mirrored in the current study, where some participants described misalignment in communication styles, the presence of conflict, and incompatibility in their relationships with other autistic people.

Another finding unique to our study is the labour and cognitive and emotional load of autistic–autistic relationships. While these relationships may reduce certain stressors, such as the need to mask or conform to neurotypical social norms, they are not automatically effortless. Many participants described their relationships with other autistic people as exhausting and requiring a high amount of energy to maintain. It has been well documented that many autistic individuals find relationships in general to be exhausting (Black et al., 2024; Strunz et al., 2017), but some research has found that autistic people feel less tired after spending time with fellow autistic people (e.g. Crompton, Hallett, Ropar, et al., 2020). Our results suggest that some autistic adults do find relationships with other autistic people to be tiring. This may be influenced by monotropic tendencies, as autistic social interaction is often driven by shared interests, and when such alignment is absent, engagement may feel more effortful (Bertilsdotter Rosqvist et al., 2023). Rather than reflecting a failure of interaction, this may instead indicate a lack of compatibility.

Another factor that may contribute to this issue is the intensity and depth of autistic relationships. Some autistic individuals prefer having fewer, but more intense, friendships (Sedgewick et al., 2019), which may mean these relationships carry greater emotional weight.

Autistic individuals may therefore experience difficulties dividing attention and energy across multiple relationships, with periods of reduced engagement occurring when attention is redirected towards special interests or when social energy is depleted. When differences in energy levels or needs are not understood or respected, managing relationships can become particularly challenging (Bertilsdotter Rosqvist et al., 2023).

This study appears to be the first to highlight the presence of conflict within autistic–autistic relationships. While much research has identified the benefits of these relationships (e.g. shared understanding, reduced masking, greater social ease), fewer studies have explored the potential challenges. Our findings indicate that while autistic individuals often relate deeply to one another, differences in communication styles, emotional regulation, and cognitive rigidity can sometimes lead to misunderstandings or conflict.

Although conflict in autistic–autistic relationships has not been explored in depth, some researchers have touched on the topic briefly. For example, Crompton, Hallett, Ropar, et al. (2020) mention how two of their participants reported difficulties in autistic–autistic relationships due to the hurtful nature of honest communication and the potential for autistic people to engage in unpredictable behaviour. Recently, Khaw and Vernon (2025) examined how partner neurotype influences romantic relationship satisfaction among autistic adults. They found that while autistic–autistic dyads experienced fewer communication challenges than other dyad types, nearly half of autistic–autistic dyads continued to experience difficulties with communication, conflict avoidance, and conflict resolution. Therefore, taken together with the present findings, emerging evidence suggests that autistic–autistic relationships, while often experienced as supportive and affirming, can also involve challenges. This highlights the need for further research that examines both their strengths and complexities.

In addition, Gillespie-Smith et al. (2024) suggest reframing the Double Empathy Problem as a continuum rather than a binary divide. Rather than viewing communication difficulties as arising solely between autistic and non-autistic individuals, they propose a spectrum of neurocultural learning and understanding, in which miscommunication can occur both within and between neurotypes. This perspective aligns with our findings, suggesting that autistic individuals can misunderstand each other due to differences in experiences and social understanding, just as misunderstandings can occur between autistic and non-autistic people. While a shared neurotype can foster deep connections, these relationships are not always seamless, and breakdowns can still occur.

We found that compatibility beyond shared neurotype was important to many participants. This is notable, as much of the existing research exploring autistic–autistic relationships has primarily emphasised shared autism status as a key factor in compatibility. Our findings extend beyond this by highlighting the importance of other factors such as physical proximity, shared interests, and personality traits. Previous research has also noted that autistic individuals with other marginalised identities may seek connections with those who share these other identities (Watts et al., 2025). For example, non-White autistic individuals may experience racial discrimination that may not be fully recognised or understood by White autistic individuals (Crompton et al., 2022). This highlights the heterogeneity of the autistic community, as relationship compatibility is influenced by multiple factors beyond a shared neurotype and cannot be solely determined by an individual’s diagnostic status (Khaw & Vernon, 2025).

### Limitations

Several limitations should be considered when interpreting these findings. First, the majority of autistic adults who completed the survey were 18–50 years of age and able to read, write, and complete the survey independently. Participants were able to complete the survey with help from a support person; however, only one participant elected to do so. Therefore, the findings from this study may not transfer to the wider autistic population including older age groups or those with co-occurring intellectual disability. Second, we encountered a high number of bot-generated and fraudulent responses. While cleaning the data, it is possible that some legitimate responses were removed if they appeared suspicious. However, we are confident that the qualitative responses analysed in this study came from genuine participants, as the excluded responses lacked meaningful qualitative data. All included responses were thoroughly examined to ensure their legitimacy and passed multiple Qualtrics security measures. Finally, all participants were based in either Australia or New Zealand, so findings may not represent autistic adults living outside of these countries.

### Implications for Future Research

Our results contribute to the small but growing body of qualitative literature exploring autistic adults’ perceptions and preferences in their relationships with other autistic individuals. While research on the benefits of autistic–autistic relationships, particularly in relation to the Double Empathy Problem, is expanding, there is significantly less work examining the potential challenges, labour, and energy demands of these relationships. Our findings suggest that while autistic–autistic relationships offer many benefits, they are also nuanced and not always effortless. Future research should further investigate these challenges, including constrains on accessibility and connection, to develop a deeper understanding of their complexities, multifaceted nature, and impact. In addition, it would also be valuable to explore how autistic individuals manage their energy levels (often referred to as “spoons”) and how they prioritise this limited resource in their relationships and daily lives.

## Conclusion

Overall, our study indicates that autistic adults have a range of perceptions and experiences regarding their relationships with other autistic people. While many positive aspects of these relationships were reported, such as a deep sense of understanding, acceptance, authenticity, and lack of judgement, there were also challenges within these relationships. Our results suggest that while autistic–autistic relationships are beneficial for a variety of reasons, these relationships, like all relationships, are complex and nuanced.

## Supplemental Material

sj-docx-1-aut-10.1177_13623613261451898 – Supplemental material for “I Accept Them, They Accept Me, We Enjoy Our Time Together”: Autistic Adults’ Preferences and Perceptions of Relationships With Other Autistic People Supplemental material, sj-docx-1-aut-10.1177_13623613261451898 for “I Accept Them, They Accept Me, We Enjoy Our Time Together”: Autistic Adults’ Preferences and Perceptions of Relationships With Other Autistic People by Hannah Minnell, Hannah Waddington, Phoebe Jordan, Beth Noble and Chris J. Bowden in Autism

## Notes

1. In this article, we use the term “non-autistic” to refer to anyone who is not autistic. However, we use the term “neurotypical” when it has been explicitly used by other authors in the context of their work.

2. “Spoons” is a metaphor for energy reserves commonly used by autistic individuals.
