# Supplementary material for: “I Accept Them, They Accept Me, We Enjoy Our Time Together”: Autistic Adults’ Preferences and Perceptions of Relationships With Other Autistic People
Source: Autism. 2026 Jun 25;30(8):2000–14. doi: 10.1177/13623613261451898 (PMC13392157; doi:10.1177/13623613261451898)
Supplement: sj-docx-1-aut-10.1177_13623613261451898 – Supplemental material for “I Accept Them, They Accept Me, We Enjoy Our Time Together”: Autistic Adults’ Preferences and Perceptions of Relationships With Other Autistic People [file sj-docx-1-aut-10.1177_13623613261451898.docx]

Q18 Do you consent to taking part in this survey?

- Yes
- No

End of Block: Introduction

Start of Block: Screening questions

Q1 Are you an adult (18 years or older) who identifies as autistic, whether through an official diagnosis or self-identification?

- Yes
- No

| Page Break |  |
| --- | --- |

Q2 What country are you currently living in?

- New Zealand
- Australia
- Other

| Page Break |  |
| --- | --- |

End of Block: Screening questions

Start of Block: Discontinue message

Q96 Thank you very much for your interest in this study. At this time, we are specifically looking for autistic adults over the age of 18 years living in Australia or New Zealand. Unfortunately, the answers you provided suggest that you do not meet our current criteria for participation. We appreciate and thank you for your time.

End of Block: Discontinue message

Start of Block: Survey overview

Q125 This survey is made up of 4 sections. They are as follows: 1 - Demographic questions 2 - Questions about your current relationships with other autistic people 3 - Questions about your views on forming new relationships with other autistic people 4 - Questions about past relationships you may have wanted to form with other autistic people when you were a child or teenager (this section is **optional**)

End of Block: Survey overview

Start of Block: Demographic questions

Q17 The following section will ask you about your demographic information

| Page Break |  |
| --- | --- |

Q3 How old are you?

- 18-24
- 25-30
- 31-35
- 36-40
- 41-45
- 46-50
- 51-55
- 56-60
- 61-65
- 65+
- Prefer not to say

Q4 How would you describe your gender? (e.g., woman, man, non-binary, gender queer, gender fluid etc.)

- Please specify: __________________________________________________
- Prefer not to say

Q136 How would you describe your sexuality? (e.g., heterosexual/straight, lesbian, gay/homosexual, bisexual, pansexual, a sexual, queer etc.)

- Please specify: __________________________________________________
- Prefer not to say

Q5 What is your ethnicity? (Please select all that apply to you)

- Australian European
- New Zealand European
- New Zealand Māori
- Aboriginal or Torres Strait Islander
- Samoan
- Cook Islands Māori
- Tongan
- Niuean
- Chinese
- Indian
- Vietnamese
- Other e.g., Japanese, Tokelauan (Please state): __________________________________________________
- ⊗Prefer not to say

Q128 Are you officially diagnosed, or self-identified as autistic?

- Officially diagnosed
- Self-identified
- Prefer not to say

Q131 At what age, approximately, were you diagnosed as autistic? (Or, if you are self-identified, how old were you when you worked this out?)

- Approximate age I was diagnosed/identified as autistic: __________________________________________________
- I don't know
- Prefer not to say

Q9 Do you have any additional diagnoses? (Please select all that apply to you)

- ⊗No other diagnoses
- Anxiety
- Depression
- Other mental health issue/s (please specify): __________________________________________________
- Attention Deficit Hyperactivity Disorder (ADHD)
- Chronic health problems
- Physical disability
- Other disability (please specify); __________________________________________________
- Other diagnosis (please specify): __________________________________________________
- ⊗I don't know
- ⊗Prefer not to say

Q10 What is the highest level of formal education you have completed?

- Primary/Intermediate School
- College/High School
- Trade/technical/vocational training
- Bachelor’s/Undergraduate University Degree
- Postgraduate University Degree
- Other (Please describe): __________________________________________________
- Prefer not to say

Q11 Are you currently in paid employment?

- Yes
- No
- Prefer not to say

Q12 What is your current employment status? (please select all that apply to you)

- Employed in a casual or part-time role
- Employed in a full-time role
- Working in a remote or telecommunicating role
- Working in a hybrid role (involving both remote work and in-office work)
- Working in an in-office/on-site role
- Working multiple jobs
- Self-employed
- Other (please specify): __________________________________________________
- ⊗Prefer not to say

Q13 What is your current occupation? (please select all that apply to you)

- Volunteering casually or part-time
- Volunteering full time
- Studying
- Stay at home parent/caregiver
- Retired
- Currently unemployed
- Unable to work due to disability/health reasons
- Other (please specify): __________________________________________________
- ⊗Prefer not to say

| 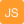 |
| --- |

Q14 Is there anything else you would like to tell us about your background that will help us understand you more?

- Yes (please specify): __________________________________________________
- No

End of Block: Demographic questions

Start of Block: Current relationships questions

Q36 The following section will ask about your current relationships with other autistic people

| Page Break |  |
| --- | --- |

Q15 Do you currently have relationships with other people who are autistic? (These people may be officially diagnosed, self-identified, or highly likely to be autistic but don't know it yet/are undiagnosed) *"Relationships" could refer to friendships, colleagues, romantic relationships etc.*

- Yes
- No
- I don't know
- Prefer not to say

| Page Break |  |
| --- | --- |

Q19 Please describe the nature of these relationships (please select all that apply to you)

- Acquaintances
- Employment colleagues
- Volunteering colleagues
- Mentoring/support relationships
- Friendships (e.g., close, childhood, special interest, hobby, study/university, online)
- Romantic relationships
- Other (please specify): __________________________________________________
- ⊗I don't know
- ⊗Prefer not to say

| Page Break |  |
| --- | --- |

Q29 Do you live with your romantic partner/s?

- Yes
- No
- Kind of/some of the time
- Prefer not to say

| Page Break |  |
| --- | --- |

Q21 Thinking of the acquaintance relationships you have with other autistic people, generally how satisfied are you with these?

- Dissatisfied
- Somewhat dissatisfied
- Neither satisfied nor dissatisfied
- Somewhat satisfied
- Satisfied
- I don't know/prefer not to say

Q23 Thinking of the employment relationships you have with other autistic people, generally how satisfied are you with these?

- Dissatisfied
- Somewhat dissatisfied
- Neither satisfied nor dissatisfied
- Somewhat satisfied
- Satisfied
- I don't know/prefer not to say

Q24 Thinking of the volunteering relationships you have with other autistic people, generally how satisfied are you with these?

- Dissatisfied
- Somewhat dissatisfied
- Neither satisfied nor dissatisfied
- Somewhat satisfied
- Satisfied
- I don't know/prefer not to say

Q25 Thinking of the mentoring/support relationships you have with other autistic people, generally how satisfied are you with these?

- Dissatisfied
- Somewhat dissatisfied
- Neither satisfied nor dissatisfied
- Somewhat satisfied
- Satisfied
- I don't know/prefer not to say

Q26 Thinking of the friendships you have with other autistic people, generally how satisfied are you with these?

- Dissatisfied
- Somewhat dissatisfied
- Neither satisfied nor dissatisfied
- Somewhat satisfied
- Satisfied
- I don't know/prefer not to say

Q27 Thinking of the romantic relationships you have with other autistic people, generally how satisfied are you with these?

- Dissatisfied
- Somewhat dissatisfied
- Neither satisfied nor dissatisfied
- Somewhat satisfied
- Satisfied
- I don't know/prefer not to say

Q75 If you like, you can tell us more about why you selected your responses. If you don't want to do this, press the next arrow **This question is optional and not included in the estimated time limit**

________________________________________________________________

________________________________________________________________

________________________________________________________________

________________________________________________________________

Q76 Thinking of all the platonic/non-romantic relationships you selected above, what are the different ways you have connected with these autistic people in the last 3 months (please select all that apply to you)

- Meeting to do an activity or have a meal/drink together
- Meeting to do separate activities in the same place
- Being part of the same sports team/activity group/club
- Taking part in a mentoring programme
- Volunteering together
- Through cultural/iwi activities
- Texting/messaging/emailing
- Online gaming
- Other (please specify): __________________________________________________
- ⊗I don't know
- ⊗Prefer not to say

Q91 If you like, you can tell us more about why you selected your responses **This question is optional and not included in the estimated time limit**

________________________________________________________________

________________________________________________________________

________________________________________________________________

________________________________________________________________

________________________________________________________________

Q78 Thinking of your romantic relationship/s, what are the different ways you have connected with these autistic people in the last 3 months (please select all that apply to you)

- Meeting to do an activity or have a meal/drink together
- Meeting to do separate activities in the same place
- Being part of the same sports team/activity group/club
- Taking part in a mentoring programme
- Volunteering together
- Through cultural/iwi activities
- Being intimate
- Texting/messaging/emailing
- Online gaming
- Other (please specify): __________________________________________________
- ⊗I don't know
- ⊗Prefer not to say

Q92 If you like, you can tell us more about why you selected your responses **This question is optional and not included in the estimated time limit**

________________________________________________________________

________________________________________________________________

Q37 On average, how often do you connect with the autistic people you have platonic/non-romantic relationships with?

- More than once a week
- Weekly
- Fortnightly
- Monthly
- Less than once a month
- It varies a lot
- Other (please specify): __________________________________________________
- I don't know
- Prefer not to say

Q94 If you like, you can tell us more about why you selected your responses **This question is optional and not included in the estimated time limit**

________________________________________________________________

________________________________________________________________

________________________________________________________________

________________________________________________________________

________________________________________________________________

Q41 On average, how often do you connect with the autistic people you have romantic relationships with?

- More than once a week
- Weekly
- Fortnightly
- Monthly
- Less than once a month
- It varies a lot
- Other (please specify): __________________________________________________
- I don't know
- Prefer not to say

Q82 If you like, you can tell us more about why you selected your responses. If you don't want to do this, press the next arrow **This question is optional and not included in the estimated time limit**

________________________________________________________________

________________________________________________________________

________________________________________________________________

________________________________________________________________

________________________________________________________________

Q132 If you like, you can tell us more about why you selected your responses. If you don't want to do this, press the next arrow **This question is optional and not included in the estimated time limit**

________________________________________________________________

________________________________________________________________

________________________________________________________________

Q135 Is there anything you would like to tell us about the other relationships you indicated that you have with autistic people?

________________________________________________________________

________________________________________________________________

________________________________________________________________

________________________________________________________________

________________________________________________________________

| Page Break |  |
| --- | --- |

Q61 Would you like to improve the quality of any relationships you currently have with other autistic people? *Improving the quality of your relationships could mean: • Having stronger attachments • Having better quality communication • Having more frequent communication • Spending more time together • Seeing each other more often*

- Yes
- Somewhat
- No
- I don't know
- Prefer not to say

| Page Break |  |
| --- | --- |

Q97 If you like, you can tell us more about why you selected your responses. **This question is optional and not included in the estimated time limit**

________________________________________________________________

________________________________________________________________

________________________________________________________________

________________________________________________________________

________________________________________________________________

Q67 Why would you like to improve the quality of the relationships you have with other autistic people?

________________________________________________________________

________________________________________________________________

________________________________________________________________

________________________________________________________________

________________________________________________________________

Q89 Why would you somewhat like to improve the quality of the relationships you have with other autistic people?

________________________________________________________________

________________________________________________________________

________________________________________________________________

________________________________________________________________

________________________________________________________________

Q86 Why would you not like to improve the quality of the relationships you have with other autistic people?

________________________________________________________________

________________________________________________________________

________________________________________________________________

________________________________________________________________

________________________________________________________________

End of Block: Current relationships questions

Start of Block: Block 9

Q124 What is this survey about?

- Shopping malls
- Favourite animals
- Autistic relationships
- Choosing restaurants

End of Block: Block 9

Start of Block: Future relationships questions

Q45 The following section will ask about your views on forming future relationships with other autistic people

| Page Break |  |
| --- | --- |

Q43 Are you interested in forming relationships with other autistic people?

- Yes
- No
- I don't know
- Prefer not to say

| Page Break |  |
| --- | --- |

Q44 Are you interested in forming more relationships with other autistic people?

- Yes
- No
- I don't know
- Prefer not to say

| Page Break |  |
| --- | --- |

Q46 Please specify the types of relationships you would like to have with other autistic people (please select all that apply to you)

- Acquaintances
- Employment colleagues
- Volunteering colleagues
- Mentoring/support relationships
- Friendships (e.g., close, special interest, hobby, study/university, online)
- Romantic relationships
- Other (please specify): __________________________________________________
- ⊗I don't know
- ⊗Prefer not to say

| Page Break |  |
| --- | --- |

Q50 How interested are you in making new acquaintances with other autistic people?

- Somewhat interested
- Interested
- Very interested
- I don't know/prefer not to say

Q70 How interested are you in making new employment relationships with other autistic people?

- Somewhat interested
- Interested
- Very interested
- I don't know/prefer not to say

Q71 How interested are you in making new volunteering relationships with other autistic people?

- Somewhat interested
- Interested
- Very interested
- I don't know/prefer not to say

Q51 How interested are you in making new mentoring/support relationships with other autistic people?

- Somewhat interested
- Interested
- Very interested
- I don't know/prefer not to say

Q52 How interested are you in making new friendships with other autistic people?

- Somewhat interested
- Interested
- Very interested
- I don't know/prefer not to say

Q53 How interested are you in having romantic relationships with other autistic people?

- Somewhat interested
- Interested
- Very interested
- I don't know/prefer not to say

Q83 If you like, you can tell us more about why you selected your responses. If you don't want to do this, press the next arrow **This question is optional and not included in the estimated time limit**

________________________________________________________________

________________________________________________________________

Q55 How would you like to connect with other autistic people? (Please select all that apply to you)

- Meeting to do an activity or have a meal/drink together
- Meeting to do separate activities in the same place
- Being part of the same sports team/activity group/club
- Taking part in a mentoring programme
- Volunteering together
- Through cultural/iwi activities
- Being intimate
- Texting/messaging/emailing
- Online gaming
- Other (please specify): __________________________________________________
- ⊗I don't know
- ⊗Prefer not to say

Q100 If you like, you can tell us more about why you selected your responses **This question is optional and not included in the estimated time limit**

________________________________________________________________

________________________________________________________________

________________________________________________________________

________________________________________________________________

Q56 At any one time, how many autistic people would you like to connect with?

- One
- Two
- Three
- More than three
- It would vary/depend
- Other (please specify): __________________________________________________
- I don't know
- Prefer not to say

Q101 If you like, you can tell us more about why you selected your responses **This question is optional and not included in the estimated time limit**

________________________________________________________________

________________________________________________________________

________________________________________________________________

________________________________________________________________

________________________________________________________________

Q57 Approximately how often would you like to connect with other autistic people platonically/non-romantically? Or, if you aren't sure, how often would be the most you would like to connect?

- More than once a week
- Weekly
- Fortnightly
- Monthly
- Less than once a month
- It would vary/depend
- Other (please specify): __________________________________________________
- I don't know
- Prefer not to say

Q102 If you like, you can tell us more about why you selected your responses **This question is optional and not included in the estimated time limit**

________________________________________________________________

________________________________________________________________

________________________________________________________________

________________________________________________________________

________________________________________________________________

Q58 Approximately how often would you like to connect with other autistic people romantically? Or, if you aren't sure, how often would be the most you would like to connect?

- More than once a week
- Weekly
- Fortnightly
- Monthly
- Less than once a month
- It would vary/depend
- Other (please specify): __________________________________________________
- I don't know
- Prefer not to say

Q84 If you like, you can tell us more about why you selected your responses. If you don't want to do this, press the next arrow **This question is optional and not included in the estimated time limit**

________________________________________________________________

________________________________________________________________

________________________________________________________________

________________________________________________________________

________________________________________________________________

| Page Break |  |
| --- | --- |

| 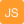 |
| --- |

Q60 Why would you like to form more relationships with other autistic people? *(For example, to reduce loneliness, share special interests, learn from each other, have someone to do hobbies with, feel more understood, find a community)*

________________________________________________________________

________________________________________________________________

________________________________________________________________

________________________________________________________________

________________________________________________________________

Q62 Why would you not like to form relationships with other autistic people at the moment?

________________________________________________________________

________________________________________________________________

________________________________________________________________

________________________________________________________________

________________________________________________________________

Q85 If you like, you can tell us more about why you selected your responses. If you don't want to do this, press the next arrow **This question is optional and not included in the estimated time limit**

________________________________________________________________

________________________________________________________________

________________________________________________________________

________________________________________________________________

________________________________________________________________

End of Block: Future relationships questions

Start of Block: Past relationships questions

Q72 The following section will ask about past relationships you may have wanted to form with other autistic people when you were a child or teenager This section is optional and is not included in the estimated completion time Would you like to answer the following section?

- Yes
- No

| Page Break |  |
| --- | --- |

Q73 Did you have relationships with other autistic people when you were a child or teenager? (These people may be officially diagnosed, self-identified, or highly likely to be autistic but don't know it yet/are undiagnosed)

- Yes
- No
- I don't know
- Prefer not to say

Q74 Thinking back to when you were a child or teenager, would you have wanted to form more relationships with other autistic people?

- Yes
- No
- It depends
- I don't know
- Prefer not to say

Q76 Thinking back to when you were a child or teenager, would you have wanted to form relationships with other autistic people?

- Yes
- No
- It depends
- I don't know
- Prefer not to say

Q127 If you like, you can tell us more about why you selected your responses. If you don't want to do this, press the next arrow **This question is optional and not included in the estimated time limit**

________________________________________________________________

________________________________________________________________

________________________________________________________________

________________________________________________________________

________________________________________________________________

| Page Break |  |
| --- | --- |

Q75 Who would you have wanted to form relationships with?

- Other autistic children/teenagers
- Autistic adults
- Both autistic children/teenagers and autistic adults
- I don't know
- Prefer not to say

Q130 If you like, you can tell us more about why you selected your responses **This question is optional and not included in the estimated time limit**

________________________________________________________________

________________________________________________________________

________________________________________________________________

________________________________________________________________

________________________________________________________________

Q77 What types of relationships would you have wanted to form with other autistic children/teenagers when you were a child or teenager? (Please select all that apply to you)

- Acquaintances
- Workplace relationships
- Volunteering relationships
- Mentoring/support relationships
- Friendships (e.g., close, school friendships, special interest friendships, hobby friendships, online friendships)
- Romantic relationships
- Other (please specify): __________________________________________________
- ⊗I don't know
- ⊗Prefer not to say

Q106 If you like, you can tell us more about why you selected your responses **This question is optional and not included in the estimated time limit**

________________________________________________________________

________________________________________________________________

________________________________________________________________

________________________________________________________________

________________________________________________________________

Q81 How would you have wanted to connect with other autistic children/teenagers when you were a child or teenager? (Please select all that apply to you)

- Meeting to do an activity or have a meal/drink together
- Meeting to do separate activities in the same place
- Being part of the same sports team/activity group/club
- Taking part in a mentoring programme
- Volunteering together
- Through cultural/iwi activities
- Being intimate
- Texting/messaging/emailing
- Online gaming
- Other (please specify): __________________________________________________
- ⊗I don't know
- ⊗Prefer not to say

Q107 If you like, you can tell us more about why you selected your responses **This question is optional and not included in the estimated time limit**

________________________________________________________________

________________________________________________________________

________________________________________________________________

________________________________________________________________

Q84 At any one time, how many autistic children/teenagers would you have wanted to connect with when you were a child or teenager?

- One
- Two
- Three
- More than three
- Other (please specify): __________________________________________________
- I don't know
- Prefer not to say

Q108 If you like, you can tell us more about why you selected your responses **This question is optional and not included in the estimated time limit**

________________________________________________________________

________________________________________________________________

________________________________________________________________

________________________________________________________________

________________________________________________________________

Q86 On average, how often would you have wanted to connect with other autistic children/teenagers when you were a child or teenager?

- More than once a week
- Weekly
- Fortnightly
- Monthly
- Less than once a month
- I don't know
- Prefer not to say

Q109 If you like, you can tell us more about why you selected your responses **This question is optional and not included in the estimated time limit**

________________________________________________________________

________________________________________________________________

________________________________________________________________

________________________________________________________________

________________________________________________________________

| Page Break |  |
| --- | --- |

Q88 Why would you have wanted to form more relationships with other autistic children/teenagers when you were a child or teenager? *(For example, to reduce loneliness, share special interests, learn from each other, have someone to do hobbies with, feel more understood, find a community)*

________________________________________________________________

________________________________________________________________

________________________________________________________________

________________________________________________________________

________________________________________________________________

| Page Break |  |
| --- | --- |

Q79 What relationships would you have wanted to form with autistic adults when you were a child or teenager? (Please select all that apply to you)

- Acquaintances
- Workplace relationships
- Volunteering relationships
- Mentoring/support relationships
- Friendships (e.g., close, special interest, hobby, online)
- Other (please specify): __________________________________________________
- ⊗I don't know
- ⊗Prefer not to say

Q111 If you like, you can tell us more about why you selected your responses **This question is optional and not included in the estimated time limit**

________________________________________________________________

________________________________________________________________

________________________________________________________________

________________________________________________________________

________________________________________________________________

Q83 How would you have wanted to connect with autistic adults when you were a child or teenager? (Please select all that apply to you)

- Meeting to do an activity or have a meal/drink together
- Meeting to do separate activities in the same place
- Being part of the same sports team/activity group/club
- Taking part in a mentoring programme
- Volunteering together
- Through cultural/iwi activities
- Texting/messaging/emailing
- Online gaming
- Other (please specify): __________________________________________________
- ⊗I don't know
- ⊗Prefer not to say

Q123 If you like, you can tell us more about why you selected your responses **This question is optional and not included in the estimated time limit**

________________________________________________________________

________________________________________________________________

________________________________________________________________

________________________________________________________________

________________________________________________________________

Q85 At any one time, how many autistic adults would you have wanted to connect with when you were a child or teenager?

- One
- Two
- Three
- More than three
- Other (please specify): __________________________________________________
- I don't know
- Prefer not to say

Q112 If you like, you can tell us more about why you selected your responses **This question is optional and not included in the estimated time limit**

________________________________________________________________

________________________________________________________________

________________________________________________________________

________________________________________________________________

________________________________________________________________

Q87 On average, how often would you have wanted to connect with autistic adults when you were a child or teenager?

- More than once a week
- Weekly
- Fortnightly
- Monthly
- Less than once a month
- I don't know
- Prefer not to say

Q113 If you like, you can tell us more about why you selected your responses **This question is optional and not included in the estimated time limit**

________________________________________________________________

________________________________________________________________

________________________________________________________________

________________________________________________________________

________________________________________________________________

| Page Break |  |
| --- | --- |

Q89 Why would you have wanted to form more relationships with autistic adults when you were a child or teenager?  *(For example, to reduce loneliness, share special interests, learn from each other, have someone to do hobbies with, feel more understood, find a community)*

________________________________________________________________

________________________________________________________________

________________________________________________________________

________________________________________________________________

________________________________________________________________

| Page Break |  |
| --- | --- |

Q134 If you like, you can tell us more about why you selected your responses **This question is optional and not included in the estimated time limit**

________________________________________________________________

________________________________________________________________

________________________________________________________________

________________________________________________________________

________________________________________________________________

| Page Break |  |
| --- | --- |

Q90 Thinking back to when you were a child or teenager, would you have wanted to improve the quality of any of the relationships you had with other autistic people? (These could be children/teenagers or adults) *Improving the quality of your relationships could mean:  • Having stronger attachments  • Having better quality communication  • Having more frequent communication  • Spending more time together  • Seeing each other more often*

- Yes
- Somewhat
- No
- I don't know
- Prefer not to say

| Page Break |  |
| --- | --- |

Q82 If you like, you can tell us more about why you selected your responses. If you don't want to do this, press the next arrow **This question is optional and not included in the estimated time limit**

________________________________________________________________

________________________________________________________________

________________________________________________________________

________________________________________________________________

________________________________________________________________

| Page Break |  |
| --- | --- |

Q92 Why would you have wanted to improve the quality of the relationships you had with other autistic people when you were a child or teenager?

________________________________________________________________

________________________________________________________________

________________________________________________________________

________________________________________________________________

________________________________________________________________

Q83 Why would you have wanted to somewhat improve the quality of the relationships you had with other autistic people when you were a child or teenager?

________________________________________________________________

________________________________________________________________

________________________________________________________________

________________________________________________________________

________________________________________________________________

Q94 Why would you have not wanted to improve the quality of your relationships with other autistic people when you were a child or teenager?

________________________________________________________________

________________________________________________________________

________________________________________________________________

________________________________________________________________

________________________________________________________________

| Page Break |  |
| --- | --- |

Q126 Thank you for taking the time to respond. Is there anything else you would like to share before ending this survey? If you have nothing else to share, press the next arrow

________________________________________________________________

________________________________________________________________

________________________________________________________________

________________________________________________________________

________________________________________________________________

| Page Break |  |
| --- | --- |

Q95 Did someone help you complete this survey (e.g., a family member, support worker, or other trusted adult?)

- Yes
- No

| Page Break |  |
| --- | --- |

End of Block: Past relationships questions

Start of Block: Raffle draw

Q87 Would you like to go in the draw to win one of 20 NZD$25 Prezzy Virtual Cards?

- Yes
- No

End of Block: Raffle draw
